# Validation of the effects of TGF-β1 on tumor recurrence and prognosis through tumor retrieval and cell mechanical properties

**DOI:** 10.1186/1475-2867-14-20

**Published:** 2014-03-03

**Authors:** Tsung-Hsien Wu, Yu-Wei Chou, Pei-Hung Chiu, Ming-Jer Tang, Chun-Wen Hu, Ming-Long Yeh

**Affiliations:** 1 Institute of Biomedical Engineering, National Cheng Kung University, No.1 University Road, Tainan City 701, Taiwan; 2 Institute of Physiology, National Cheng Kung University, Tainan, Taiwan

**Keywords:** Cell mechanical properties, TGF-β1, EMT, Angiogenesis, Tumor-recurrence, Cancer-metastasis

## Abstract

**Background:**

*In vivo*, the transforming growth factor-beta1 (TGF-β1)-induced epithelial to mesenchymal transition (EMT) occurs in seconds during cancer cells intravasation and extravasation. Although it has been established that cellular stiffness can change as a cancer cell transformed, the precise relationship between TGF-β1-induced mesenchymal stem cell mechanics and cancer prognosis remains unclear. Accordingly, it is hard to define the effects of EMT on cell mechanical properties (CMs), tumor recurrence and metastasis risks. This study bridges physical and pathological disciplines to reconcile single-cell mechanical measurements of tumor cells.

**Methods and results:**

We developed a microplate measurement system (MMS) and revealed the intrinsic divergent tumor composition of retrieval cells by cell stiffness and adhesion force and flow cytometry analysis. After flow cytometry sorting, we could measure the differences in CMs of the Sca-1^+^-CD44^+^ (mesenchymal-stem-cell-type) and the other subgroups. As well as the stiffer and heterogeneous compositions among tumor tissues with higher recurrence risk were depicted by MMS and atomic force microscopy (AFM). An *in vitro* experiment validated that Lewis lung carcinoma (LLC) cells acquired higher CMs and motility after EMT, but abrogated by SB-505124 inhibition. Concomitantly, the CD31, MMP13 and TGF-β1 enriched micro-environment in the tumor was associated with higher recurrence and distal lung metastasis risks. Furthermore, we report a comprehensive effort to correlate CMs to tumor-prognosis indicators, in which a decreased body weight gain ratio (BWG) and increased tumor weight (TW) were correlated with increased CMs.

**Conclusions:**

Together, we determined that TGF-β1 was significantly associated with malignant tumor progressing. In terms of clinical applications, local tumor excision followed by MMS analysis offers an opportunity to predict tumor recurrence and metastasis risks.

## Background

Lung cancers are among the most prevalent types of cancer in humans and are responsible for approximately 13.8% and 27.7% of annual cancer cases and related deaths, respectively [1]. The vicious progression of cancer is highly related to the epithelial-to-mesenchymal transition (EMT) [2]. Since 1994, transforming growth factor-beta1 (TGF-β1) has been known to be an effective EMT inducer and thus plays a key role in the early process of cancer cell metastasis [3,4]. TGF-β1 can mediate a diverse range of cellular responses, including the suppression of cell proliferation [5], cytoskeleton rearrangement and phosphorylation [6] as well as the disruption of cell-cell junctions [7]. The active TGF-β1 has been shown increase mesenchymal stem-like cells (MSCs), whereas inhibition of TGF-β1 activity prevents the development of MSCs with self-renewing and tumor-initiating capacities [8,9]. There are limited tools can identify the MSCs and TGF-β1 activation, however, the increased cell stiffness is one remarkable mechanical characteristic of TGF-β1 induced EMT [10].

Recently, the cell mechanical properties (CMs) have been proposed as an indicator of multiple cellular processes, including cancer malignant transformation [11], metastasis [12-15] and apoptosis [16,17]. Of the promising diagnostic methods, compressive stiffness might possibly be used to predict the onset of leukostasis [18]. With magnetic tweezers, tensile stiffness phenotypes could be measured and used to grade the metastatic potential of tumor cells [19]. Change in cell stiffness is a new characteristic of cancer cells that affects the way they spread [20,21]. The importance of CMs to cancer is appreciated, yet the contributions of CMs to tumor recurrence and prognosis remain unclear.

Moreover, primary tumor excision is the standard therapy for early defined tumors [22]. Unfortunately, some patients with seemingly localized tumors eventually die of disseminated disease. Additionally, most studies report recurrence rates of 11% for T1 tumors and 25% for T2 tumors that were treated with local excision alone [23,24]. The prognosis of metastatic pulmonary tumors has been reported to be regulated by various factors such as the organs in which primary tumor is located, the number of metastatic foci or excised tumors and the tumor doubling time [25]. Only a few studies have sporadically reported on prognostic factors in cases of pulmonary metastasis. Hence, the ability to examine prognostic factors from retrieved tumors with an accurate diagnosis method is needed.

Accordingly, we endeavored to establish a microplate measurement system (MMS) to investigate the CMs of tumor-retrieved cells, especially the MSCs type, from tumor-bearing mice. Eventually, we examined the correlation between CMs and several tumor prognosis indicators. This could benefit cancer diagnostic investigations by providing predictable mechanical properties of tumor recurrence and prognosis.

## Results

### Flow cytometry identification of tumor-retrieved cells

**Figure 1:**
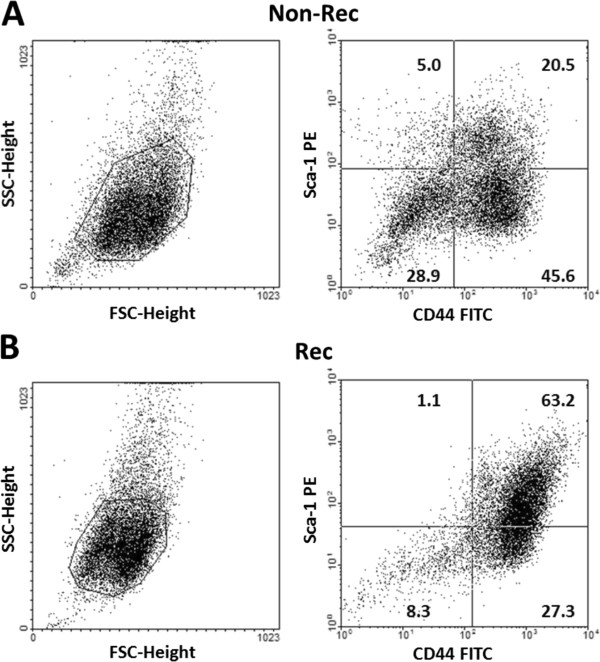
**Immunophenotypic profiles of tumor-retrieved cells.** The tumor-retrieved cells from mice were labeled with PE-conjugated Sca-1 and FITC-conjugated CD44 antigens and analyzed by flow cytometry. From the **(A)** non-recurrence (Non-Rec) and **(B)** recurrence (Rec) group, the cells that were positive for CD44 and Sca-1 were gated and then further analyzed. The percentages of gated Sca-1^-^-CD44^-^, Sca-1^+^-CD44^-^, Sca-1^-^-CD44^+^ and Sca-1^+^-CD44^+^ cells in total tumor cells are presented on plot. All of the tumor-retrieved cells were sorted into four subgroups and cultured separately for later analyses.

Flow cytometry was performed on cells that were retrieved from first-time excised primary tumors in mice. We assigned the cells into two groups according to the tumor recurrence status, either non-recurrence (Non-Rec) or recurrence (Rec). All tumor-retrieved cells were collected from 10 different tumor-bearing mice, where 5 mice were in the Non-Rec group and the other 5 mice were in the Rec group. The tumor-retrieved cells were characterized by their immunophenotypic profiles via flow cytometry. Based on the gating parameters, we noted that the tumor-retrieved cells were mainly composed of CD44^+^ Lewis lung carcinoma (LLC) cells (Figure 1). Flow cytometry analysis also revealed that the Sca-1^+^-CD44^+^ subgroup occupied 20.5% of the total cells from the Non-Rec group (Figure 1A) and 63.2% from the Rec group (Figure 1B). Additionally, the percentage of the Sca-1^-^-CD44^-^ subgroup in the Non-Rec group (28.9%) was higher than in the Rec group (8.3%). The Sca-1^+^-CD44^-^ subgroup was scarce (5.0 and 1.1%) while the Sca-1^-^-CD44^+^ subgroup was more enriched (45.6 and 27.3%) in both the Non-Rec and Rec groups.

### Tumor retrieval CMs

To analyze the mechanical properties of single tumor-retrieved cells, we choose isolated cells for microscopic observations and measurements under a 40× objective. We excluded the cells that early detached from the concanavalin A (con-A) coated backplate of cantilever, where the tensile mechanical properties were not fully measured. To obtain a calibration scale, 4.8 pixels/μm, standard grid images were taken using the same objective with a CCD camera (TCA-10, Tucsen, China).

**Figure 2:**
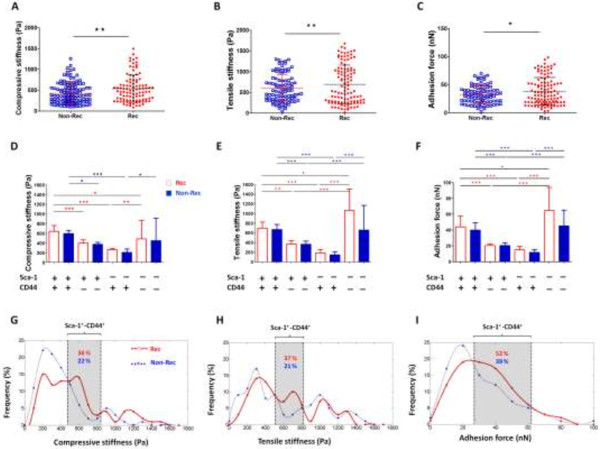
**CM measurements of the tumor-retrieved cells with different recurrence status before and after flow-cytometry sorting.** All cells were assessed with the microplate mechanical measurement system (MMS). The **(A)** compressive stiffness (CS), **(B)** tensile stiffness (TS) and **(C)** adhesion force (AF) of the non-recurrence (Non-Rec) and recurrence (Rec) group were measured and graphed as the mean (dotted line) ± SD (upper and lower index) in the scatter plots. *, p < 0.05; **, p < 0.01. **(D, E and F)** The red and blue bars represent the Rec and Non-Rec groups, respectively. After sorting, each subgroup of cells was maintained separately for CM measurements. Based on the data, the double positive subgroup had higher CMs than the single positive subgroups, and a greater variation was observed in the double negative subgroup. **(G, H and I)** The distributions of the CM properties after Gaussian curve fit. Red dotted lines indicate the Rec group, and blue dashed lines indicate the Non-Rec group. The gray areas contain the suspected mesenchymal stem cells (MSCs), which are Sca-1^+^-CD44^+^. The number of peaks were counted after all CM data were fitted, and noticeably only one peak is present in each of the gray areas from the Rec population.

Data from a total of 228 cells were collected from 10 different tumor-bearing mice, where 118 cells were in the Non-Rec group and the other 110 cells were in the Rec group. The average compressive stiffness (CS), tensile stiffness (TS) and adhesion force (AF) were significantly higher in the Rec group (CS: 539.1 ± 32.7 Pa, TS: 693.4 ± 44.9 Pa and AF: 38.5 ± 2.3 nN) than in the Non-Rec group (CS: 428.7 ± 22.9 Pa, TS: 601.8 ± 34.6 Pa and AF: 32.5 ± 1.7 nN) by 111 Pa (25%), 92 Pa (15%) and 6 nN (19%), respectively (Figure 2A,B and C; Table 1). Therefore, the MMS resolution (2 nN) was sufficient to distinguish a difference in CMs between the groups.

**Table 1:** **The mechanical properties of tumor-retrieved cells and *in vitro *** **cultured LLC cells under no treatment, TGF-β1 treatment and SB-505124 + TGF-β1 co-treatment**

**Tumor-retrieval**		***In vitro***	
Non-Rec		Control	
CS (Pa)	428.7 ± 22.9 (N = 118)	CS (Pa)	384.9 ± 15.4 (N = 12)
TS (Pa)	601.8 ± 34.6 (N = 92)	TS (Pa)	530.5 ± 9.9 (N = 14)
AF (nN)	32.58 ± 1.7 (N = 88)	AF (nN)	27.45 ± 3.2 (N = 9)
AFM (Pa)	758.1 ± 134.8 (N = 54)		
Rec		TGF-β1	
CS (Pa)	539.1 ± 32.7 (N = 98)**	CS (Pa)	465.5 ± 29.2 (N = 13)**
TS (Pa)	693.4 ± 44.9 (N = 108)**	TS (Pa)	593.7 ± 42.6 (N = 14)**
AF (nN)	38.56 ± 2.3 (N = 110)*	AF (nN)	54.04 ± 9.1 (N = 15)***
AFM (Pa)	1095.0 ± 176.6 (N = 50)**		
		SB-505124 + TGF-β1	
		CS (Pa)	404.9 ± 15.4 (N = 18)
		TS (Pa)	560.5 ± 9.9 (N = 18)
		AF (nN)	33.5 ± 3.2 (N = 12)

*CS*, Compressive stiffness; *TS*, Tensile stiffness; *AF*, Adhesion force; *AFM*, Atomic force microscopy-measured stiffness. Each value represents the mean ± standard deviation (n = cell number for analysis). *P < 0.05; **P < 0.01; ***P < 0.001.

Noticeably, the measured CMs of the four subpopulations of tumor-retrieved cells had a discrete distribution, which was indicative of heterogenic mechanical properties. After sorting by flow cytometry, we obtained four subgroups (Sca-1^+^-CD44^+^, Sca-1^+^-CD44^-^, Sca-1^-^-CD44^+^ and Sca-1^-^-CD44^-^) of tumor-retrieved cells from the Non-Rec and Rec group. In the Rec group, the average CS values for the Sca-1^+^-CD44^+^, Sca-1^+^-CD44^-^ and Sca-1^-^-CD44^+^ subgroups were 641.3 ± 21.2 Pa, 397.4 ± 12.9 Pa and 264.3 ± 4.8 Pa, respectively; in the Non-Rec group, the corresponding values were 595.3 ± 11.8 Pa, 373.8 ± 8.2 Pa and 207.9 ± 17.3 Pa (Figure 2D,E and F; Table 2). Based on the MMS measurements, the double positive Sca-1^+^-CD44^+^ subgroup exhibited a higher average CS, TS and AF than the single positive subgroups in both the Rec and the Non-Rec cell populations. However, no significant differences were found between the Non-Rec and Rec groups for all CMs. The CM values of the double negative Sca-1^-^-CD4^-^ subgroup showed significant variation among the cells, as indicated by large standard deviations ranging from 36% to 54%.To evaluate the distribution of cells with different CMs, we performed Gaussian curve fits of all the CMs data (Figure 2G,H and I). Noticeably, there are more peaks on the Rec group curves than on the Non-Rec group curves in general (number of peaks, Rec vs. Non-Rec: 4 vs. 2 for CS and 4 vs. 3 for TS). For each CM property, at least one subpopulation of cells was enriched in the Rec group but scarce in the Non-Rec group.

**Table 2:** The mechanical properties of tumor-retrieved cells, which were sorted into four subgroups by flow cytometry

**Non-Rec**		**Rec**	
Sca-1^+^-CD44^+^		Sca-1^+^-CD44^+^	
CS (Pa)	595.3 ± 11.8 (N = 32)	CS (Pa)	641.3 ± 21.2 (N = 32)
TS (Pa)	674.0 ± 19.2 (N = 29)	TS (Pa)	700.3 ± 31.1 (N = 17)
AF (nN)	39.6 ± 1.4 (N = 29)	AF (nN)	43.8 ± 1.9 (N = 17)
Sca-1^+^-CD44^-^		Sca-1^+^-CD44^-^	
CS (Pa)	373.8 ± 8.2 (N = 26)***	CS (Pa)	397.4 ± 12.9 (N = 26)*
TS (Pa)	366.6 ± 12.3 (N = 31)**	TS (Pa)	373.4 ± 14.6 (N = 23)***
AF (nN)	20.2 ± 0.8 (N = 21)***	AF (nN)	20.2 ± 0.5 (N = 21)***
Sca-1^-^-CD44^+^		Sca-1^-^-CD44^+^	
CS (Pa)	207.9 ± 17.3 (N = 17)***	CS (Pa)	264.3 ± 4.8 (N = 18)***
TS (Pa)	149.1 ± 17.9 (N = 14)***	TS (Pa)	190.2 ± 13.2 (N = 28)***
AF (nN)	11.6 ± 0.8 (N = 14)***	AF (nN)	14.8 ± 1.2 (N = 12)***
Sca-1^-^-CD44^-^		Sca-1^-^-CD44^-^	
CS (Pa)	446.8 ± 165.5 (N = 50) *	CS (Pa)	485.3 ± 265.5 (N = 34)
TS (Pa)	660.1 ± 283.7 (N = 37) *	TS (Pa)	1066.0 ± 364.4 (N = 47)
AF (nN)	45.1 ± 24.6 (N = 29) *	AF (nN)	64.5 ± 26.4 (N = 31)

*CS*, Compressive stiffness; *TS*, Tensile stiffness; *AF*, Adhesion force. Each value represents the mean ± standard error (cell number for analysis). *P < 0.05; **P < 0.01; ***P < 0.001.

We then determined if the nature of the cells, mesenchymal stem-like cells (MSCs), could be correlated to the CMs, using data from the individual subgroups. The regions that correspond to the Sca-1^+^-CD44^+^ subgroup, which stand for MSCs, are indicated on the Gaussian curves for the Non-Rec cells (average values of 595.3 ± 11.8 Pa, 674.0 ± 19.2 Pa and 39.6 ± 1.4 nN for CS, TS and AF, respectively) and the Rec cells (average values of 641.3 ± 21.2 Pa, 700.3 ± 31.1 Pa and 43.8 ± 1.9 nN for CS, TS and AF, respectively) (Figure 2D,E and F). The average values of the CMs for the Sca-1^+^-CD44^+^ cells are located within the gray areas on the frequency distribution graphs (Figure 2G,H and I), which indicate the presence of a single population of MSCs. The total percentages of cells from the Non-Rec group and from the Rec group within the gray areas were 22% and 36% based on CS distribution, 21% and 37% based on TS distribution and 39% and 52% based on AF distribution, respectively. Our cell mechanical analysis concluded that the distribution of the CM values reflected the higher percentage of Sca-1^+^-CD44^+^ cells, which were likely MSCs, in the Rec population compared with the Non-Rec population. We suggest that cell mechanical properties can be used to predict the presence of MSCs in tumors.

### AFM measurements of stiffness

**Figure 3:**
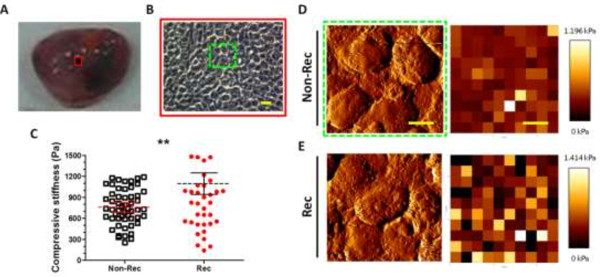
**AFM force-mappings of freshly prepared frozen tumor slices. (A)** Image of the excised tumor. **(B)** Phase-contrast image of the central part of the tumor. The freshly prepared frozen tumor tissue slices were assessed by AFM. The compressive stiffness of **(C)** and the error-signal images and force-mapping of **(D)** Non-Rec tumor and **(E)** Rec tumors are presented. Scale bar = 10 μm. **, p < 0.01.

We used atomic force microscopy (AFM) to measure micron-level stiffness in the tumor tissues (Figure 3A and B). The mean stiffness of the Non-Rec tumors was 758 ± 134.8 Pa, whereas the Rec tumors were significantly stiffer with an average of 1095 ± 176.6 Pa (Figure 3C; Table 1; *p <* 0.01). The force-mappings revealed that greater amounts of heterogeneous and stiffer components existed in the Rec than Non-Rec tumors (Figure 3D and E), although we could not identify which component contributed to tumor progression and stiffening based on the AFM data alone.

### Assessment of tumor malignancy based on CD31, MMP13 and TGF-β1 expression

**Figure 4:**
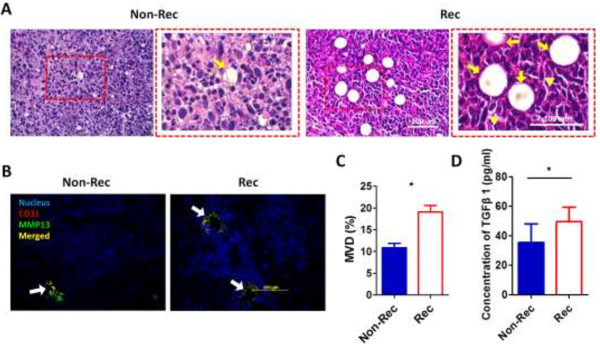
**Assessment of tumor malignancy based on CD31, MMP13 and TGF-β1 expression. (A)** H&E staining of sliced tissue samples from Non-Rec and Rec tumors. Areas containing angiogenic features are demarcated by the red lines and indicated with yellow arrows. The yellow arrow-heads indicated the elongated fibroblast. An enlarged view of the angiogenic area is shown to the right of each sample. **(B)** Immunofluorescent staining of Non-Rec and Rec tumor tissue samples for CD31 and MMP13. Colocalized staining of CD31 and MMP13 is indicated by the white arrows. **(C)** The bar graph demonstrates the difference in microvascular density (MVD) between the groups. **(D)** The ELISA assay showed that TGF-β1 was expressed in a recurrence-status-dependent manner, because the level of released TGF-β1 was lower in the culture medium from the Non-Rec group than from the Rec group. Scale bar = 100 μm in the H&E images; Scale bar = 200 μm in the Immunofluorescent images. *, p < 0.05.

Immunohistochemical observations of tumor sections identified relatively fewer microvessels within the Non-Rec tumors than within the Rec tumors (Figure 4A). The crucial role of MMP-13 in angiogenesis promotion and maintenance was supported by the localization of MMP-13 near CD31, which is a known marker of endothelial cells in newly formed blood vessels (Figure 4B). CD31 expression was circularly aligned with the vessels, and the microvascular density (MVD) of the Rec tumors was significantly higher (1.9-fold; p < 0.05) than that of the Non-Rec tumors (Figure 4C). The level of autocrinally released TGF-β1 was significantly different between the Non-Rec group and the Rec group by 75% (Figure 4D).

### *In vitro* treatment with SB-505124 abrogates TGF-β1-induced EMT and changes in E-cadherin expression, cell motility and cell mechanics

**Figure 5:**
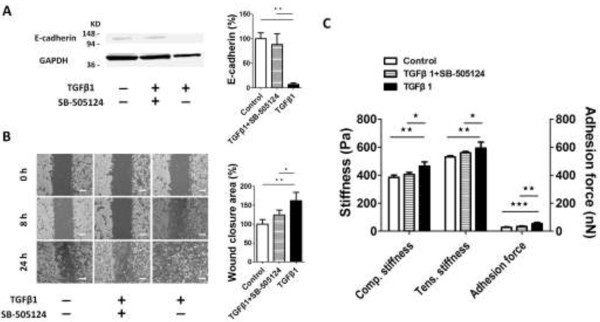
***In vitro *treatment with SB-505124 abrogates TGF-β1-induced EMT and changes in E-cadherin expression, cell motility and cell mechanics. (A)** The SB-505124 + TGF-β1-treated LLC group consisted of cells that were treated with 10 ng/ml TGF-β1 in the presence of 10 μM SB-505124 for 48 hr. Cell lysates were analyzed by western blot analysis with an anti-E-cadherin antibody. Equal loading of protein was verified by the analysis of GAPDH. This experiment was repeated three times with similar results. The data showed that TGF-β1-treated LLC cells expressed less E-cadherin than the untreated cells and the SB-505124 + TGF-β1 co-treated cells. E-cadherin was equally expressed in the latter two samples. **(B)** Phase contrast images of the wound-healing assay are shown. The migration of the cell monolayer was observed by time-lapse microscopy and photographed at 0, 8 and 24 h after wounding. Original magnification was 10×. The percentages of wound closure for the control, the SB-505124 + TGF-β1-treated and the TGF-β1-treated cells were calculated from the mean of four wound widths at each time point. The average closure rate was highest in TGF-β1-treated cells. **(C)** LLC cells were assessed with the MMS. TGF-β1-treated cells displayed significant differences in stiffness and adherence when compared with the control and the SB-505124 + TGF-β1 co-treated cells based on CS, TS and AF measurements. Scale bar = 100 μm. *, p < 0.05, **, p < 0.01, ***, p < 0.001.

To examine the effect of SB-505124 and TGF-β1 co-treatment on Lewis lung carcinoma (LLC) cells, we first noted that TGF-β1 treatment alone reduced the expression of the junctional E-cadherin protein by 94% in the LLC cells. Interestingly, SB-505124 treatment reversed TGF-β1-induced downregulation of E-cadherin in LLC cells (Figure 5A).In line with the change in E-cadherin expression, we observed a functional increase in cell motility after TGF-β1 treatment. A 24 hr wound-healing assay revealed that the wound-closure rate of TGF-β1-treated cells that had undergone EMT was 1.5 fold of the rate of the control cells. Exposure to SB-505124 blocked the accelerated motility of EMT cells (Figure 5B).

A total of 14 control, 15 TGF-β1-treated and 18 SB-505124 + TGF-β1-treated LLC cells were measured by MMS. Interestingly, the post-EMT cells had a significantly higher resistance to external compressive and tensile stresses than the control cells (increments of 81 Pa (21%) and 63 Pa (12%) in CS and TS, respectively; p < 0.05). Additionally, the adhesion force increased markedly (27 nN; 100%) after EMT. However, there were no significant differences between the control cells and the SB-505124 + TGF-β1-co-treated cells for all CMs (Figure 5C; Table 1).

### Metastatic and invasive abilities of tumor-retrieved cells

**Figure 6:**
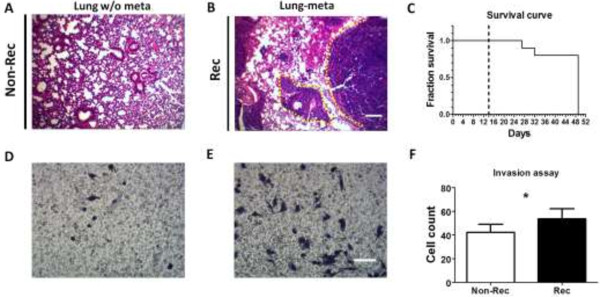
**Metastatic and invasive abilities of tumor-retrieved cells. (A, B)** Representative H&E stainings of mouse lung biopsies. **(A)** Subjects from the Non-Rec group were sacrificed at day 49, and no metastatic areas were found in the lungs. **(B)** Obvious tumor metastases were found in the lungs of spontaneously deceased subjects in the Rec group, as marked by the yellow dotted lines. **(C)** The survival curve demonstrated that 2 subjects were lost due to poor prognosis and lung metastasis, and the remaining subjects were sacrificed at day 49. The tumors from all subjects were excised at day 14 (primary, black dotted line). **(D, E)** Representative phase contrast images of the matrigel invasion assay. The Non-Rec group **(D)** had fewer invading cells than the Rec group **(E)**. **(F)** Quantification of the invasion assay results. The difference between the 2 groups is statistically significant. Scale bars represent 100 μm and 20 μm in the H&E and invasion images, respectively. *, p < 0.05.

We removed the lung tissues and grossly observed their integrity. Metastatic features were not seen in the Non-Rec sample (Figure 6A). However, metastatic nodules were identified in the lung biopsies from 2 of the Rec group (Figure 6B). Noticeably, lung metastasis was observed in 2 subjects from the Rec group that had died on day 27 and 32 (Figure 6C). *In vitro* invasion assays were performed on collagen matrigel-coated inserts, and the migratory cell number was 26.6% less in the Non-Rec group (42.2 ± 6.9 counts) than in the Rec group (53.6 ± 8.5 counts; p < 0.05; Figure 6D,E and F).

### Association between CMs and tumor prognosis indicators

**Figure 7:**
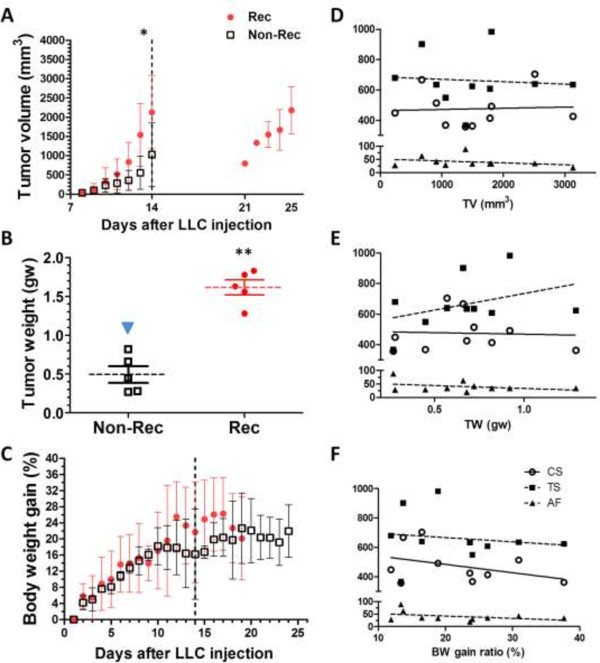
**Association between CMs and tumor prognosis indicators. (A)** Tumor volume (TV) estimates were plotted against time, and the dotted line represents the day of the tumor excision. The TVs of the recurrent tumors were calculated after day 21. **(B)** The tumor weight (TW) of the Rec group was significantly higher than that of the Non-Rec group. **(C)** The body weight gain (BWG) data of all subjects were plotted against time. **(D, E, F)** The regression analyses of CS (circle), TS (filled square) and AF (filled triangle) versus TV **(D)**, TW **(E)** and BWG **(F)** are shown. *, p < 0.05; **, p < 0.01.

We routinely recorded the tumor dimensions and estimated the tumor volume (TV). The TV of both groups progressively increased at a different rate. After LLC injected for 14 days, the average TV of tumors from the Rec group (1540.07 ± 814.54 mm^3^) were significantly larger (2.8-fold) than those from the Non-Rec group (559.82 ± 431.35 mm^3^; p < 0.05). After tumors excision, we allowed recurrent tumors to develop and noted that the TV increased drastically after day 21 (Figure 7A). The tumor weight (TW) of the Rec group (1.62 ± 0.21 gw) was 2.2-fold over that of the Non-Rec group (0.49 ± 0.24 gw; p < 0.01; Figure 7B). There were no significant differences in the body weight gain ratio (BWG) between the groups. Furthermore, we observed that the BWG of the Rec group fluctuated after day 12 and declined after day 17. Noticeably, a growth plateau appeared at day 19 in the Non-Rec group (Figure 7C).

The relationships between the tumor prognosis markers including TV, TW and BWG and the CMs measured by MMS were shown in Figure 7D,E and F. There was no significant correlation between TV and the CMs (Figure 7D; Table 3). In contrast, TW correlated positively with tensile stiffness (r = 0.15; Figure 7E; Table 3), while BWG correlated negatively with compressive stiffness (r = -0.15), tensile stiffness (r = -0.02) and adhesion force (r = -0.15) (Figure 7F; Table 3).

**Table 3:** Association between CMs and tumor prognosis indicators

		**Tumor prognosis indicators**		
		**TV**	**TW**	**BWG**
**Cell mechanical properties**	CS	0.01	- 0.01	- 0.15^*^
	TS	- 0.01	0.15^*^	- 0.02
	AF	- 0.09	- 0.11^*^	- 0.15^*^

The bold text with star symbol is used to indicate a statistical association but low.

## Discussions

### Flow cytometry identification of tumor-retrieved cells

We hypothesized that if MSCs were involved in tumor recurrence and metastasis to the lung from the primary tumor, a Sca-1^+^-CD44^+^ population of cells should be present in the primary tumors. The flow cytometry analysis showed that the cells that were retrieved from the Rec tumors had a higher percentage of the Sca-1^+^-CD44^+^ subpopulation than the cells from the Non-Rec tumors (Figure 1). Sca-1^+^-CD44^+^ cells have already been shown to have a mesenchymal stem cell-like profile [26,27], to be enriched for genes that are involved in cell motility, proliferation and angiogenesis and to be associated with decreased patient survival and EMT [26,28]. Comparable percentages of the Sca-1^+^-CD44^+^ subgroup were observed between the Rec and Non-Rec groups (63.2 vs. 20.5%), suggesting that this subgroup of cells played a role in tumor recurrence.

Sca-1, which stands for stem cell antigen-1, is a glycosyl phostidylinositol-anchored cell surface protein that is associated with both stem cell and progenitor cell activities as well as with tumor initiating potential [29]. CD44, a hyaluronic acid receptor, is a multifunctional class I transmembrane glycoprotein. CD44 is also one of the most commonly studied cell surface markers, which is expressed by almost every type of cancer cells [30]. Thus, the high percentage of the CD44^+^ subgroup in the Non-Rec and Rec populations indicated that cancer cells constituted the major part of the tumors. Moreover, the low percentage of the Sca-1^+^-CD44^-^ subgroup in both populations implied a minor representation by non-cancerous progenitor cells. The cells without Sca-1 and CD44 expression were stromal cells, such as fibroblasts and endothelial cells. Using flow cytometry, we sorted the tumor-retrieved cells into four subgroups and further analyzed the specific role of the Sca-1^+^-CD44^+^ MSCs in tumor progression.

### Tumor retrieval CMs

As indicated by the flow cytometry analysis, the two pools of tumor-retrieved cells from Non-Rec and Rec tumors contained not only LLC cells but also fibroblasts and endothelial cells and MSCs. Thus, our CM data had wide variations. Despite the heterogeneity of cells, we were able to differentiate the cells with different recurrence potential based on the CMs. We found that cells with higher stiffness and adhesion force were more likely to form recurrent tumors.

The complex composition of the tumor-retrieved cell populations should not mask the ability of certain tumor cells to undergo additional transformation to form recurrent tumors or metastases. In general, cancer cells are typically soft and more pliant than their healthy counterparts [12,17,31-34]. Paradoxically, biophysical analyses have revealed that single cancer cells at any period of tumor development may acquire increased stiffness after TGF-β1-induced EMT, as was described for adenocarcinoma [35] and sarcoma cells [36]. However, many cancer biologists believe that EMT is required for tumor cells to leave the primary tumor and metastasize [37], which suggests that these stiffer cells would need to gain the abilities to invade into the surrounding tissues and bear higher stress [35].

The factors that affect CM measurement may be confounded by the mechanical heterogeneity of cell populations and, in the case of tumor cell studies, asynchronous malignant status. Thus, we used an established method [38] to purify subgroups of cells by flow cytometry. We compared the CMs among the Sca-1^+^-CD44^+^, Sca-1^+^-CD44^-^ and Sca-1^-^-CD44^+^ subgroups and searched for correlations with the cellular malignant potential. The CM values of the Sca-1^+^-CD44^+^ cells, which were likely MSCs, were significantly higher than those of the Sca-1^+^-CD44^-^ and Sca-1^-^-CD44^+^ subgroups, which contained the non-cancerous progenitor cells and the non-progenitor cancer cells, respectively (Figure 2D,E and F). As we know, MSCs play an important role in cancer cell extravasation, which is characterized by the facilitation of cancer cell adhesion and retention on vessel walls [39]. We assumed that the increases in stiffness and adhesion force in the Sca-1^+^-CD44^+^ subgroup were due to EMT that was induced by endogenous TGF-β1 in the tumors. Taken together, these results suggest that high stiffness and adherence force are characteristic of cells expressing mesenchymal stem cell markers with high tumorigenic potential, and that these cells are responsible for tumor malignancy and metastasis. The combination of flow cytometry and cell mechanical measurements may serve as a tool for evaluating and sensing biological and biomechanical changes in tumor cells, which could be clinically relevant.

Interestingly, the CM values of the Sca-1^-^-CD44^-^ subgroup had large standard deviations. This result was most likely due to the complex composition of this subpopulation, which contained cells such as fibroblasts and endothelial cells, the stiffness of which has been previously calculated to be 1.4-6.8 kPa [40].

According to the flow cytometry analysis and the MMS measurements (Figure 1 and 2G,H and I), the percentages of Sca-1- or CD44-expressing cells in the Non-Rec and Rec groups correlated with the distribution of the CM values. The Sca-1^-^-CD44^+^ subgroup, which contained the non-progenitor cancer cells, was more represented in the Non-Rec group than in the Rec-group (45.6% for Non-Rec vs. 27.3% for Rec) based on flow cytometry analysis, and the CM profiles also reflected this difference, where Non-Rec group had a higher percentage of cells with <500 Pa stiffness and <25 nN adhesion force (the ‘soft’ cells) than the Rec-group. The rationale of the tumor-retrieved CMs possessed tumor prognosis responsiveness was the differentiation of specific MSCs subgroup. Those cells had CM values between those of the soft cancer cells and the stiff stromal cells, specifically with CS at 500–800 Pa, TS at 500–800 Pa and AF at 25–60 nN (Figure 2G,H and I). Changes in the mechanical properties of cancer cells may affect the way they migrate, invade and disseminate.

### AFM measurements of stiffness

In this study, MMS and AFM used different scales of cell mechanical measurements, where MMS was performed on single cells and AFM was performed on tissues. Both methods revealed a positive correlation between tumor CMs and recurrence risk. Additionally, due to the measurement scale difference, the compressive stiffness that was detected by AFM was approximately 2-fold of the measurements by MMS (Table 1). It is known that tumor-derived ECM is biochemically distinct in its composition from normal ECM. Furthermore, it has been shown that tumor stroma (∼400 Pa) is typically stiffer than normal stroma (∼150 Pa), and that breast cancer tissue in particular could be tenfold stiffer than normal breast tissue (150 Pa for breast cancer stroma vs. 1.5 kPa for normal breast tissue) [41-43]. During cancer metastasis, the angiogenic process brings different types of cells close to each other, and each cell type may possess a different degree of stiffness. For example, endothelial cell layers are typically at ~1.2 kPa, and stromal tissues, including fibroblasts or smooth muscle cells, are typically at ~5 kPa [44]. Indeed, cancer cell dissemination is regulated in part by stiffness, and cancer cells could often encounter stiffness gradients within a tumor (durotaxis), which can guide cell migration.

One clinical similarity with the above observation is that clinicians often diagnose tumors based on differences in tissue rigidity, which are sensed by palpation. Tissue stiffness can reflect the presence and the potential malignancy of a tumor, yet the relevance of tissue stiffness to tumor pathogenesis has been largely ignored. A previous study found that even a small increase in matrix rigidity could perturb tissue architecture and enhance tumor growth by inducing Rho-generated cytoskeletal tension to promote focal adhesion assemblies and increase ERK activation [45]. Thus, tensional homeostasis might be essential to oncogene-driven ERK activation and could induce cytoskeletal contractility to enhance integrin-dependent growth and destabilize tissue architecture [46]. As such, conditions that induce tissue fibrosis (matrix stiffening) [47] or situations that amplify oncogene activity could facilitate malignant transformation by increasing cell contractility.

This study was conceived because tissue palpation in the clinic inspired us to investigate if cell mechanics might be indicators for cancer malignancy. In summary, the combined MMS and AFM results depicted precisely graded increments in the magnitude of CMs as the cells emerged in the stiffer tumor microenvironment. These altered cell-ECM interactions precede and can even contribute to malignant transformation, such as the case in leukemia [18], and the ECM in many solid tumors is typically significantly stiffer than normal ECM [45]. Moreover, cells are known to become attuned to the specific tissue in which they reside [48].

### Assessment of tumor malignancy based on CD31, MMP13 and TGF-β1 expression

The increased angiogenesis in the Rec group tumors was revealed by both H&E and immunofluorescent staining. We also noted that CD31-expressing endothelial cells occupied the sites of angiogenesis (Figure 4A and B). Tumor angiogenesis contributes to tumor cell proliferation and metastasis by supplying oxygen and nutrients and removing cellular wastes [49,50].

Previously studies have reported that a decrease in the stiffness of endothelial cells may account for the breakdown of the endothelial barrier function, suggesting that biomechanical alterations are sufficient to facilitate the transmigration and invasion of cancer cells into the surrounding stroma [51]. It has indeed been shown that invasive cancer cells may diminish the endothelial barrier function by shedding the cell-cell adhesion receptors from the cell surface in the presence of MMPs [51]. In this study, we also observed the colocalization of CD31 and MMP13 in the tumor tissues (Figure 4B).

MMPs are a family of proteolytic enzymes that can degrade ECM components. They are thus functionally important to malignancy-associated tissue rearrangements. Stromal MMP-13 expression is known to be required for the invasion and metastases of breast cancer and melanoma [52,53]. Impaired tumor growth and metastasis have also been shown in MMP-13-deficient mice, which could be attributed to reduced angiogenesis [53]. Our data suggest that host MMP-13 is responsible for blood vessel formation and infiltration into tumor mass.

In this study, we found a higher concentration of TGF-β1 in the culture medium of the Rec tumor retrieved cells than in the medium of the Non-Rec tumor retrieved cells (Figure 4D), suggesting that TGF-β1 might play a role in promoting tumor recurrence. Previous studies have shown that TGF-β1 could stimulate connective tissue formation, angiogenesis and other changes that may favor invasion and metastasis in an autocrine [52,54] and/or paracrine [55,56] manner. Tumor cells can activate a stromal response that is further amplified after MMP-digested stroma releases TGF-β1 [57]. Thus, TGF-β1 production might be associated with MMP activity during metastasis [58]. Shipitsin and colleagues have shown that TGF-β1 and the TGF-β type I receptor are overexpressed in CD44^+^ cells within breast tumors [28]. TGF-β1 is a potent inducer of EMT in mammary cells, and its overexpression has been associated with acquisition of tumor stem-like properties [59]. It has also been suggested that TGF-β1 promotes tumor invasion via its paracrine effect on tumor stroma [60,61]. Initiation of metastasis may also start with the signaling of TGF-β1.

### *In vitro* treatment with SB-505124 abrogates TGF-β1-induced EMT (EMT) and changes in E-cadherin expression, cell motility, and cell mechanics

To further investigate the role of TGF-β1 in LLC cells, we used SB-505124, which is a neutralizing antibody against TGF-β1 receptors, to block TGF-β1 activity *in vitro*. We used western blotting, wound healing assay and MMS to identify changes in protein expression, cellular motility and CMs, respectively. The delocalization of junctional E-cadherin after TGF-β1-induced EMT can be blocked by SB-505124 as previously reported [62]. Loss of cell-cell contacts, increased cell migration and invasion, and degradation of the extracellular matrix are all features of EMT [63].

Our results showed that the cells underwent EMT and acquired higher motility after TGF-β1 treatment, but the effects were blocked by SB-505124 (Figure 5B). EMT, which converts epithelial cells into motile mesenchymal cells and promotes invasive and migratory properties, plays a key role in metastasis [3,64]. Moreover, cells without E-cadherin exhibit a contact-inhibited but accelerated motility and it is a prerequisite for EMT [65].

In this study, we also confirmed that the CMs were altered after EMT. Compared with the control cells, the compressive stiffness, tensile stiffness and adhesion force of the post-EMT cells were increased by 21.1%, 11.9% and 100%, respectively (Figure 5C, Table 1). Such increments in CMs might contribute to the early stage of cancer cell metastasis [3], permitting cancer cells to disseminate into sites such as the lymphatic and blood vasculatures through EMT [64]. In an analysis of the cooperative role of TGF-β1 in tumor development, a higher concentration of autocrinally-released TGF-β1 was observed in the culture medium of the Rec cells but not in the medium of the Non-Rec cells (Figure 4D). We observed that TGF-β1 stimulation occurs in malignant cancer cells during EMT and that alterations in the CMs after EMT are critical for tumor recurrence and metastasis.

From a molecular perspective, a stronger adhesion force and self-contractile force may accelerate the motility of the EMT cells. Cytokine functions are context-dependent, which can either promote or inhibit tumor progression. At later stages of tumor progression, TGF-β1 acts as a tumor promoter. It appears that the shift from being a tumor suppressor to a tumor promoter is due to increased resistance of tumor cells to the inhibitory signals of TGF-β1 [66]. Indeed, inhibition of TGF-β receptor has been shown to reverse EMT and induce a mesenchymal-to-epithelial differentiation in CD44^+^ mammary epithelial cells [28]. These results suggest that SB-505124, as an EMT antagonist, could act as a ‘brake’ to stop the accelerating motility of cells after TGF-β1 induction through the restoration of E-cadherin expression, furthermore, to diminish the increased CMs.

### Metastatic and invasive abilities of the tumor-retrieved cells

In our study, lung metastasis were not observed eventually in all animals. However, we observed metastatic nodules in the lungs of 2 mice from Rec group that died of cancer. Our observations were not surprising because we anticipated that the tumors formed by the Rec cells had a higher likelihood of recurrence and metastasis to the lung compared with those that were formed by the Non-Rec cells. The invasion assay also revealed that the Rec cells were more invasive than the Non-Rec cells. The bilateral molecular crosstalk between the cancer cells and the stroma can be mediated through direct cell-cell contacts or secreted molecules [52,54], and this crosstalk can lead to increased invasiveness [67] and motility [68] of cancer cells. In this study, the correlation between angiogenesis and the expression of MMP13 and TGF-β1 also confirmed that the tumor microenvironment was crucial to the promotion of tumor recurrence and metastasis.

During the invasion assay in a mock 3D microenvironment, the front parts of the cells invaded through the porous membrane and then latched onto the opposite side. Meanwhile, a strong traction force was needed to drag the entire cell body through the membrane. In line with our MMS results, the cells that successfully invaded were those with increased stiffness and adhesion forces due to elevated TGF-β1expression. Our results illustrated the importance of CMs in cancer cell invasion and metastasis.

### Association between CMs and tumor prognosis indicators

Numerous studies have investigated CM changes, using methods such as micropipette aspiration [69-71], AFM [72-74] and microplate mechanical systems [70,75,76], which is now emerging as a diagnostic tool. However, whole-cell mechanical changes remained poorly documented, and the correlation between CMs and tumor prognosis was unknown.

We monitored the tumor growth daily. We observed accelerated tumor growth (TV and TW) in the tumors from the Rec group compared with the ones from the Non-Rec group. However, the BWG ratios did not differ between the groups. There was a possible correlation between the *in vivo* data and the CM measurements by MMS. Higher cell tensile stiffness was found in the cells that formed heavier tumors. It indicates that the more rigid cells were likely more malignant and therefore proliferated faster into a condensed tumor mass. All of the CM values were inversely correlated with the BWG ratio, which indicated that the CMs increased in poorly developed tumors in response to the amount of nutrients that were encroached by the tumors, leading to the decline in the BWG ratio.

## Conclusions

In this study, we defined the malignancy status (angiogenesis, TGF-β1 expression, MMP13 expression, tumor recurrence and lung metastasis) and poor-prognosis indicators (decreased BWG and increased TV and TW) that are associated with increased cell stiffness and adhesion force. We concluded that early expression of endogenous TGF-β1 affected the mechanical properties of tumor cells as well as tumor growth, angiogenesis and metastasis. Reciprocally, increased cell stiffness and adhesion force can enhance the cell-environment contact and crosstalk. We found that the Sca-1^+^-CD44^+^ cells were likely candidates for disseminated or metastatic cells, and this subpopulation was present at different percentages in the populations of cells that were retrieved from the Non-Rec and Rec tumors. Our results were also consistent with the predictions under the mesenchymal stem cell hypothesis. We have shown that tumor recurrence parallels the development of metastasis and that cells expressing mesenchymal stem cell markers may be important for lung metastasis.

The stiffness of leukemia and ovarian cancer cells has been recently used as a diagnostic marker and a marker for chemotherapeutic response [18,19,31]. Our data also indicate that CM properties could be used as potential clinical biomarkers. MMS is an effective and feasible tool to detect mechanical changes in cells, which can then be used to predict tumor cell malignancy. Therefore, MMS can provide novel evidence for clinical diagnoses and therapeutic effect assessments at the cellular level.

## Methods

### Xenograft mouse model and *ex vivo* tumor-retrieved cells

The murine lung cancer cell line (Lewis lung carcinoma, LLC1, BCRC 60050), derived from C57/BL6 mice, was purchased from the Bioresource Collection and Research Center (BCRC, Taiwan). To validate the effects of TGF-β1 induced EMT on CMs, *in vitro* cultured LLC were treated with 10 ng/ml recombinant human TGF-β1 (PeproTech, London, United Kingdom) in 1% FBS-DMEM for a 48 hr induction. The TGF-β1 receptor kinase inhibitor, SB-505124, was purchased from Sigma (Sigma, USA). SB-505124 is a specific inhibitor of TGF-β superfamily type I receptors ALK4 (activin receptor-like kinase 4), ALK5, and ALK7 [9]. Cells were then treated with TGF-β in the presence of SB-505124 (10 μM) for a total of 2 days.

To construct the xenograft tumor-bearing mice model, LLC cells were inoculated into C57/BL6 mice according to previously published methods [53,77,78]. Briefly, LLC (10^6^) in 100 μl PBS were injected intradermally into the flanks of 6 to 8-week-old mice. At 2 weeks post-LLC inoculation, the tumor-bearing mice were anesthetized and the tumors were removed. Subsequently, the mice were maintained for tumor-recurrence and lung-metastasis examinations (in accordance with the National Cheng-Kung University Animal Center standards; affidavit of approval of animal use protocol, approval number: 101265). At 7 weeks post-inoculation, all mice were euthanized with overdose anesthetic injections.

The tumor-retrieved cells were collected according to a modified method [79,80]. Briefly, part of the minced mouse tumor was digested in collagenase at 37°C for 3 hr and then filtered through sterile 58-nm nylon mesh (BD Bioscience, CA, USA). Collected cells were centrifuged at RT for 3 min at 800 rpm, and erythrocyte hemolysis was performed with RBC lysis buffer (10 nmol/L potassium bicarbonate, 155 mmol/L ammonium chloride, 0.1 mmol/L EDTA (pH 7.4)) for 5 min, after which the suspension was again centrifuged for 5 min at 800 rpm. The pellet was resuspended in 10 ml of fresh medium and added to a dish for incubation. Subsequently, the tumor-retrieved cells were washed and then incubated on culture dishes that were coated with fibronectin. After 2 days of incubation, the non-adherent immune cells were eliminated. Subsequently, adherent cells were harvested by trypsinization for analysis or transplantation.

### Flow cytometry analysis

Cells were washed twice with PBS and then harvested. Detached cells (10^6^ cells/100 μl) were resuspended in PBS supplemented with 0.5% fetal bovine serum. Combinations of fluorochrome-conjugated monoclonal antibodies against mouse CD44 (FITC; cat. #11-0441) and Sca-1 (PE; cat. #12-5981) (eBioscience, CA, USA) were added to the cell suspensions as recommended by the manufacturer, and the suspensions were incubated at 4°C in the dark for 20 min.

The phenotypes of cultured retrieval cells were analyzed by BD FACSaria (BD Biosciences, CA, USA) fitted with BD FACSDiva software. Followed the previous method [38], the compensation was performed using single color controls. Samples were analyzed to compare the negative selection antibodies against Sca-1-PE or CD44-FITC. Sca-1^+^-CD44^+^ selection were then gated to show percent double-positive for CD44 and Sca-1. A post sort analysis was performed to determine the purity of the retrieval cells. The labeled cells were analyzed on a FACS Calibur flow cytometer (BD Biosciences, CA, USA) according to the manufacturer’s instructions.

### Experimental design of cell mechanics measurements using MMS

**Figure 8:**
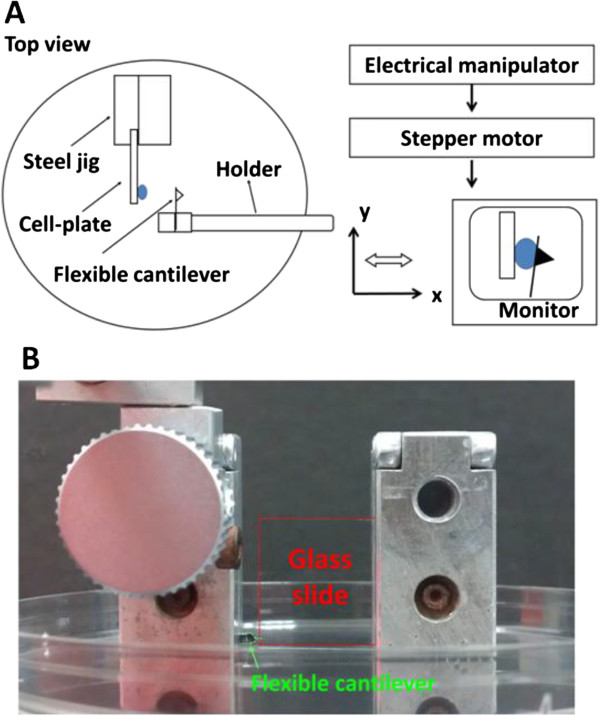
**Schema and photo image of the microplate mechanical measurement system (MMS). (A)** The MMS comprised a cell-seeding glass slide and a flexible cantilever at the opposite position. We manipulated the cantilever by inputting commands through a computer to the stepper motor, and then operated the cantilever to move along the x-axis of the images shown on the monitor. **(B)** The component locations were adjusted perpendicular to the bottom of the petri dish above the microscope.

Glass microscope slides were sterilized and coated with an extracellular substrate layer via incubation in 10 μg/cm^2^ type I rat tail collagen (BD Bioscience, CA, USA) overnight, followed by 2 washes in PBS. Suspended cells (2 × 10^5^ cells/ml) were allowed to adhere to the collagen-coated slides for 4 hr before the experiment (Figure 8A and B). Culture medium that contained 30 mM HEPES was added to the dish to prevent pH changes over the course of the experiment. The calibration scale under the 40× objective was 4.8 pixels/μm.Before the measurements, all flexible AFM cantilevers were cleaned with sulfochromic acid to remove organic compounds and were subsequently sterilized. The cleaned cantilevers were functionalized in 0.5 mg/ml concanavalin A (con-A; Sigma, California) for 30 min at room temperature. The 3D position of the AFM probe was manually adjusted to be near the glass slide surface and was parallel-aligned by verifying the microscopy images in a different focus plane than the focus drive (Figure 8A and B). The MMS resolution was calculated from the deflection of the cantilever multiplied by the calibrated spring constant. Therefore, the estimated resolution was 2 nN.

Firstly, we applied a compression force through the con-A coated flexible cantilever with a piezoelectric actuator, which was displaced to the cell by a prescribed amount (typically 5 μm) at a constant speed (0.42 μm/s). The reversed tension force followed, in which the cantilever was pulled with the cell away from collagen coated glass slide; the cantilever was deflected until the cell detached.

### Image analysis and mechanical property estimations

Each cell was recognized as either a spheroid or hemi-spheroid with rotational symmetry around the x-axis. The x- and y-dimensions were defined as the cell height and diameter, respectively. Axial strain (ϵ) was calculated as the change in cell height (measured immediately) divided by the initial cell height (measured prior to compression). Additionally, the contact area (Ac) between the cells and the cantilever was assumed to be circular because of the symmetry of the cell shape and its value was estimated from the measured cell diameter, which changed progressively during measurement. The calibrated cantilever deflection was measured by synchronizing the images. The measured force (F) was calculated with Hooke’s law from the deflection of the cantilever multiplied by the calibrated spring constant of the cantilever. An image analysis program was encoded with MATLAB software (Math Works), using the following steps: mean filtering, histogram equalization, edge filtering, edge detection, and force reduction, as performed in a previous study [81], to detect the cantilever deflection pixels and transform them into force measurements. Stress (σ) was calculated with equation (1):

(1) $$\sigma =\frac{F}{A_{c}}$$

Stiffness (E) was estimated from the stress versus the strain, using equation (2):

(2) $$E=\frac{\sigma }{\epsilon }$$

### AFM measurements of stiffness

AFM was used to identify the individual tumor stiffness that contribute to the origins of the stiffening tumor. The bottom quarters of retrieved tumors were embedded in OCT aqueous embedding compound within a disposable plastic base mold and were snap-frozen by direct immersion into liquid nitrogen as previously described [82]. Frozen tissue blocks were then cut into 20-μm sections with disposable low-profile microtome blades (Leica, 819) on a cryostat (Leica, CM1900-3-1). The excised tumor samples from 4 animals (2 Rec and 2 Non-Rec) were used for the AFM force mapping analysis. All preparative steps were performed in a sterile buffer supplemented with a protease inhibitor cocktail (Sigma, CA, USA). Mechanical manipulations were kept to a minimum at all times during sample preparation. The atomic force microscope (JPK, NanoWizard II, Germany) was set up for inverted microscopy (Zeiss Axio Observer, Germany). A pyramid cantilever (P1L450B, Nanosensors, USA) with a 1 nN-μm-sec-1 loading rate while in contact mode was used to obtain 3 different 50 × 50 μm^2^ force–volume maps over 10 × 10 point grids.

### Immunofluorescence and immunohistochemistry staining

Tumor and lung tissue immunostaining was performed as previously described [83]. A hematoxylin and eosin (H&E)-stained section was obtained from each tissue block. To evaluate tumor angiogenesis and invasiveness, tumor sections were stained with rat anti-mouse CD31 (1:100; Pharmingen, Heidelberg, Germany) and rabbit anti-mouse MMP-13 (1:500; Santa Cruz Biotechnology, Heidelberg, Germany) Subsequently, the sections were washed with PBS and incubated with an Alexa568-conjugated goat anti-rat secondary antibody (for CD31; 1:400; Dianova, Hamburg, Germany), and an Alexa488-conjugated goat anti-rabbit secondary antibody (for MMP13; 1:400; Molecular Probes, Göttingen, Germany) for 2 hr at room temperature. The nuclei were counterstained with Hoechst dye H33342 (1:1000; Calbiochem-Novabiochem, Schwalbach, Germany). The CD31, MMP13 and nucleus-conjugated fluorescent labels were excited with lasers at 568, 488 and 405 nm, respectively, and fluorescence was detected with a scanning confocal microscope (FV-1000, Olympus).

The MVD measurements were obtained simultaneously within each area. The MVD was measured according to Weidner’s method, in which all distinct vessels are counted in a high power field [84]. Briefly, MVD herein refers to the area in a tissue sample that is enclosed within the vascular space relative to the total area.

### ELISA for TGF-β1 in culture medium

The levels of TGF-β1 were determined using a TGF-β1 ELISA Kit (USCN, TX, USA) according to the manufacturer’s instructions. The lower limit for the cytokine detection was 5 pg/ml. A total of 10^5^ cells from each group of tumor-retrieved cells were seeded into each well of 12-well plate. Cells were cultured in media containing 10% FBS for 18 hr, and then 100 μl of each supernatant media was used for assay.

### Western blot for E-cadherin expression

E-cadherins expressed by control, TGF-β1 and SB-505124 + TGF-β1 treated LLC cells were examined by western blot analysis. Cells were solubilized in lysis buffer. An equal amount of each protein lysate were analyzed by western blot analysis with mouse anti-E-cadherin and anti-GAPDH polyclonal antibody (BD Biosciences, CA, USA).

### ibidi wound healing assay

By monolayer wound assay [85], we examined the effects of SB-505124 on motility of LLC cells in response to TGF-β1 induced EMT. Cells were induced EMT as before mentioned. The control, TGF-β1 and SB-505124 + TGF-β1 treated cells, were kept in serum-free medium for 24 hr. Three confluent cell groups were wounded with a μ-Dish 35-mm culture ware (ibidi GmbH, integrated BioDIagnostic). After washing, the medium was replaced by normal culture medium. Photographs of the wounded area were taken by phase–contrast microscope with 10× objective at 0, 8 and 24 hr after wounding. For evaluation of wound closure, four randomly selected points along each wound were marked and the horizontal distance of migrating cells from the initial wound was measured with Image J (version 6.0; NIH).

### Transwell invasion assay

Tumor-retrieved cells from every subject were allowed to migrate across a membrane with 8-μm pores (BD Biosciences, CA, USA) towards a medium that contained 20 μg/mL FBS at 37°C, as previously described [86]. Briefly, we first placed Transwell membrane inserts into a 24-well plate. Next, 50 μl of collagen matrigel (BD Biosciences, CA, USA), which had been diluted to 1 mg/ml, were added to the inserts. Next, we added 600 μl of DMEM that contained 20% FBS and 200 μl of prepared cells (2 × 10^4^) in DMEM containing 1% FBS to the insert. After a 16-hr incubation, the cells on the membrane were fixed and stained with methanol and Giemsa, and the cells were counted under a 10 × objective.

### Tumor prognosis indicators evaluation

The body weights and palpable tumor dimensions were periodically evaluated every 2 days for 7 weeks with a weight scale and beam caliper. To determine the tumor volume (TV), the greatest longitudinal diameter (length) and the greatest transverse diameter (width) were determined, and TV was calculated according to the modified ellipsoid formula (Length × Width^2^) [87]. At 2 weeks post-LLC cell inoculation, the excised tumor-weights (TW) were recorded. The body weight gain ratio (BWG) was calculated from the increased weight divided by the original weight of each mice.

### Data analysis

Statistical analysis was performed with SPSS version 17.0 software. First, the normal distribution was verified with the Shapiro-Wilk test. One-way ANOVA and Tukey’s post hoc test were used to examine the differences between the groups. Pearson correlation coefficients were established by examining the interrelationships between the MMS and tumor prognosis factor data from each experiment. A *r* between 0 and 0.25 was considered a low association, between 0.25 and 0.5, a fair association, between 0.5 and 0.75, a moderate association, and > 0.75, a high association [88]. The significance level was set at *p <* 0.05.

## Abbreviations

TGF-β1: Transforming growth factor-beta1; EMT: Epithelial to mesenchymal transition; CMs: Cell mechanical properties; MMS: Microplate measurement system; AFM: Atomic force microscopy; LLC: Lewis lung carcinoma cell; MSCs: Mesenchymal-stem-like cells; TV: Tumor volume; BWG: Body weight gain ratio; TW: Tumor weight; ECM: Extracellular matrix; Non-Rec: Non-recurrence; Rec: Recurrence; CS: Compressive stiffness; TS: Tensile stiffness; AF: Adhesion force; MVD: Microvascular density; H&E: Hematoxylin and Eosin.

## Competing interests

The authors declare that they have no competing interest.

## Authors’ contributions

THW designed experiments, analysed data and wrote the paper. THW built up the MMS and image analysis with programs developed with YWC. CWH and PHC carried out most of the cell and animal experimental studies. YWC participated in the AFM experiments. MJT revised the manuscript. MLY, supervised the experiments and completed the manuscript. All authors read and approved the final manuscript.
